# Litter Breakdown and Microbial Succession on Two Submerged Leaf Species in a Small Forested Stream

**DOI:** 10.1371/journal.pone.0130801

**Published:** 2015-06-22

**Authors:** Molli M. Newman, Mark R. Liles, Jack W. Feminella

**Affiliations:** Department of Biological Sciences, Auburn University, Auburn, Alabama, United States of America; Wageningen University, NETHERLANDS

## Abstract

Microbial succession during leaf breakdown was investigated in a small forested stream in west-central Georgia, USA, using multiple culture-independent techniques. Red maple (*Acer rubrum*) and water oak (*Quercus nigra*) leaf litter were incubated *in situ* for 128 days, and litter breakdown was quantified by ash-free dry mass (AFDM) method and microbial assemblage composition using phospholipid fatty acid analysis (PLFA), ribosomal intergenic spacer analysis (RISA), denaturing gradient gel electrophoresis (DGGE), and bar-coded next-generation sequencing of 16S rRNA gene amplicons. Leaf breakdown was faster for red maple than water oak. PLFA revealed a significant time effect on microbial lipid profiles for both leaf species. Microbial assemblages on maple contained a higher relative abundance of bacterial lipids than oak, and oak microbial assemblages contained higher relative abundance of fungal lipids than maple. RISA showed that incubation time was more important in structuring bacterial assemblages than leaf physicochemistry. DGGE profiles revealed high variability in bacterial assemblages over time, and sequencing of DGGE-resolved amplicons indicated several taxa present on degrading litter. Next-generation sequencing revealed temporal shifts in dominant taxa within the phylum *Proteobacteria*, whereas γ-*Proteobacteria* dominated pre-immersion and α- and β-*Proteobacteria* dominated after 1 month of instream incubation; the latter groups contain taxa that are predicted to be capable of using organic material to fuel further breakdown. Our results suggest that incubation time is more important than leaf species physicochemistry in influencing leaf litter microbial assemblage composition, and indicate the need for investigation into seasonal and temporal dynamics of leaf litter microbial assemblage succession.

## Introduction

Allochthonous (external) inputs are the major source of energy and nutrients within food webs of many small forested streams [1], with this input primarily entering streams as leaf litter from surrounding riparian vegetation [2–5]. Nutrient release by stream microorganisms during breakdown is a critical process affecting whole-stream metabolism [5–7]. Litter also provides a vital structural habitat for many stream benthic macroinvertebrate communities [8, 9]. Thus, the structural and energetic importance of leaf litter to forested streams makes it an integral part of overall ecosystem integrity and function [1, 7, 10].

In streams, leaf breakdown, defined as the decomposition of vascular plant detritus described by Webster and Benfield [1], consists of 3 primary phases—leaching, conditioning, and fragmentation. Once immersed, litter undergoes chemical leaching usually within 24 to 48h [11]. Colonization and conditioning by fungi and bacteria softens litter and facilitates further decomposition within days after immersion [12, 13]. Litter is subsequently fragmented by physical abrasion and processing by macroinvertebrate consumers (i.e., shredders) [12, 14, 15], which, in combination, accelerate breakdown [16].

Leaf physicochemical characteristics, such as initial N concentration, C:N,structural compounds (e.g. lignin, hemicellulose, cellulose), physical leaf toughness, and physical barriers (e.g. cutin) vary strongly among leaf species [11, 17, 18], and such variation can affect breakdown [11]. Ostrofsky [17] showed that the best leaf chemistry predictors of breakdown were %N, C:N, condensed tannin concentration, and %lignin:%N. Coulson & Butterfield [19] showed high N, and secondarily P, concentrations were positively correlated with microbial densities and breakdown within a bog. Studies of forest floor litter decomposition also have shown litter Ca to significantly predict breakdown rates, likely because of its effects on earthworm abundance in these systems [20]. Low C:N also is associated with increased microbial activity, often occurring in litter low in cellulose and lignin and with faster breakdown rates [21]. Several other studies reported concentrations of structural compounds (e.g., lignin, hemicellulose, and cellulose) within litter are inversely related to breakdown, possibly inhibiting fungal and bacterial colonization of litter [22–24]. Leaf toughness and cutin also are negatively correlated with leaf mass loss during breakdown, acting as potential barriers and resisting microbial degradation [18, 25].

Stream fungi and bacteria are critical to litter breakdown, and their relative contributions to leaf conditioning indicate a greater initial contribution by fungi with bacterial conditioning increasing over time [26, 27]. Given their critical role in litter breakdown and associated energy cycling, it is necessary to investigate the presence and potential functional role of specific taxa present during breakdown. In this context, data on individual taxa presence can provide insight into the specific biochemical and physiological processes involved in breakdown. For example, increased abundance of N-fixing *Nitrobacter* species in decomposing litter may indicate increased nitrification during certain stages of litter breakdown. In addition, in disturbed systems, knowing which taxa are typically present in decomposing litter under certain environmental conditions facilitates comparison within and among streams, and could inform the efficacy of stream restoration practices in re-establishing system function [28].

To date, studies usually have characterized fungal and bacterial assemblages present at different stages of breakdown using a combination of cultivation, microscopy, and assays of reproductive structures and metabolic products [29–33]. The advent of molecular techniques, including DNA sequencing and fingerprinting, provides an opportunity to characterize microbial assemblage dynamics during litter breakdown with increased resolution of microbial taxa, as well as a heightened ability to quantify assemblage similarity [34]. For example, ribotype fingerprinting techniques have been used to quantify fungal preferences of leaves during colonization [35], and the application of denaturing gradient gel electrophoresis (DGGE) to study bacterial and fungal assemblages revealed temporal shifts in microbial assemblages during conditioning [36–38]. Phylogenetic resolution of microbial taxa during breakdown would thus contribute greatly to our knowledge of stream microbial population dynamics, as well as the role of leaf physicochemistry as a potential modulator of assemblage structure during breakdown. However, many molecular techniques have known biases, including differential amplification of microbial taxa by varying primer sets, co-migration of ribotypes, or reproducibility of the denaturing gradient, which necessitates a pluralistic approach in understanding assemblage dynamics. Thus, we aimed to use several molecular methods (i.e., DGGE, RISA, PLFA, and next-generation sequencing) to quantify microbial succession during breakdown for 2 litter species of contrasting leaf physicochemistry (i.e., water oak and red maple) in a small, forested coastal plains stream, by 1) comparing differences in fungal and bacterial assemblages between leaf species and, thus, 2) characterizing bacterial taxa associated with litter during breakdown.

## Materials and Methods

### Study site

The study was conducted at Kings Mill Creek (UTM 0720701E 3600036N), a second-order, low-gradient stream at the Fort Benning Military Installation (FBMI) in west-central Georgia, USA. Authority to this field site was granted by FBMI (U.S. Army) with FBMI liaison Hugh Westbury arranging site access on sampling dates. FBMI occurs south of the Fall Line in the Sand Hills sub-ecoregion of the Southeastern Plains ecoregion [39]. Kings Mill Creek is a low-nutrient stream with sandy substrate [40], and an intact deciduous riparian canopy [41, 42] consisting mostly of red maple (*Acer rubrum*), dogwood (*Cornus* spp.), yellow poplar (*Liriodendron tulipifera*), sweetgum (*Liquidambar styraciflua*), sweetbay magnolia (*Magnolia virginiana*), black gum (*Nyssa sylcatica*), and water oak (*Quercus nigra*) [43]. The Kings Mill Creek watershed was largely forested (>85% forest cover) [40] with a high abundance of shredder macroinvertebrates (K.O. Maloney, unpubl. data), implying the importance of litter to the stream’s trophic economy.

### Experimental design

An *in situ* litter decomposition experiment was conducted using 2 leaf species, *Acer rubrum* (red maple) and *Quercus nigra* (water oak). Both species were common in riparian zones at the study site and across FBMI in general [44]. In general, red maple leaves are 2 to 4 inches long and wide, and water oak leaves are 1.5 to 4 inches long and 0.5 to 2 inches wide [45]. These species span a range of breakdown rates, with red maple having a medium breakdown rate (*k* = 0.005–0.010) and water oak a relatively low rate (*k*<0.005) (Webster & Benfield, 1986). In addition, maple species (*Acer* spp.) show a strongly contrasting chemistry compared to oaks (*Quercus* spp.), with maple having higher N content (low C:N) and oak having a higher C:N and lignin content [17]. The leaves of these two species also differ physically in that maple species often have thicker leaves and an increased specific leaf mass per unit area [46] while oak leaves possess a thick, waxy cuticle and high concentration of tannins [47].

We incubated litter *in situ* [48] over 9 collection dates (days 0, 1, 2, 4, 8, 16, 32, 64, and 128) from January to May 2007, which spanned early microbial colonization and those temporal changes occurring as litter breakdown proceeded. We established 3 leaf treatments, which included 2 single-species treatments of red maple and water oak alone and 1 mixed-litter treatment containing a 1:1 mix of red maple and water oak leaves. Leaf packs of both single species and mixed litter packs were placed in mesh bags (0.1524 m x 0.3048 m, Nylon Net Co., Memphis, TN, USA), with mesh size large enough (6.35 mm) to allow macroinvertebrate colonization on one side and a smaller (3.175 mm) mesh on the other side to reduce loss of litter particles from inside the bag during incubation. We placed leaf packs in 8 runs, which are stream microhabitats with relatively homogeneous depth and current of moderate, non-turbulent flow [49–51]. Within each run, we used a randomized complete block design with 4 blocks per run and each block containing one replicate of each treatment (S1 Fig). Runs were sampled randomly over the study with one run sampled per date. We collected leaves for leaf packs from a single tree of each species during fall 2006 (December-January) using tarps strung below trees to accumulate abscised leaves. We used single trees for each species to reduce variability in initial phyllosphere composition from cultivar-specific variations in leaf chemistry. We air-dried leaves in a sterile Class II biosafety cabinet to a constant mass, weighed into 4-g aliquots, and then placed them into sterilized mesh bags until deployed. Mixed litter packs contained 2 g of each leaf species. Once filled, mesh bags were sewn closed with nylon and then anchored in the stream with rebar. Leaf species were sampled on day 0 by immersing packs in stream water and then removing and returning them to the laboratory to quantify handling loss [11]. Day 0 packs were selected to represent the initial phyllosphere microbial assemblage for each leaf species, and these packs were treated similarly to all others for further processing.

On each date, we randomly selected one run and removed all 4 blocks of leaf packs for each treatment (n = 4/date). Leaves were placed in a Ziploc bag, and returned on ice to the laboratory. A 2-leaf subsample was removed from each leaf pack, ground in liquid N_2_ and stored at –80°C until processed for microbial assemblage characterization (below). We used only the single-species treatments for microbial assemblage characterization (below). The remaining leaves were rinsed and dried to a constant mass at 60°C, weighed, and then combusted in a muffle furnace at 550°C for 2 h. The ashed residue was weighed, and this weight was subtracted from the pre-combusted dry mass to estimate breakdown rate (as ash-free dry mass, AFDM). Breakdown rates were estimated using an exponential decay model [11] as the slope of the regression line of ln (% AFDM remaining) vs time [52].

To characterize variation in environmental conditions known to affect breakdown [1, 53] we also quantified streamwater temperature, depth, and current velocity at or near each collection point. Temperature was recorded hourly with HOBO Temp data loggers (Onset Computer Corp., Pocasset, MA, USA). We also quantified depth and current velocity within each run (n = 12/run) to assess the spatial variation in initial depth and current velocity that might have influenced microbial assemblages independently of date. Leaf pack depth was measured using a meter stick placed at the top center of each leaf pack, and a Marsh-McBirney Flow-Mate current meter (Frederick, MD, USA) was used to measure current velocity conditions at each leaf pack. In addition, current velocity inside each leaf pack was estimated by positioning an empty “dummy” bag over the probe placed immediately upstream of each leaf pack with current velocity typically ranging from ~0 to 0.10 m/s faster outside (vs. inside) a given leaf pack.

### Microbial lipids and assemblage characterization

#### Microbial lipids

Relative abundance of bacterial and fungal lipids on incubating litter was estimated by using phospholipid fatty acid (PLFA) analysis to quantify relative abundance of different lipid markers associated with bacteria and fungi present over the study [54, 55]. PLFA uses readily degraded phospholipid fatty acids to estimate the abundance of microbial biomass, and can accurately characterize lipid profiles of other benthic microbial assemblages [54, 56]. The PLFA method was adapted from Sasser [57] for saponification, formation of fatty acid methyl esters (FAMEs), extraction, and a base wash, as follows. First, we placed an approximately 680-mg sample of the liquid N_2_-ground litter in a 20 mL test tube. Samples were saponified to liberate fatty acids from lipids of lysed cells with 1.0 mL saponification reagent (45 g NaOH, 150 mL methanol, 150 mL deionized water), vortexed for 10 s, heated to 100°C for 5 min in a water bath, and then vortexed and reheated to 100°C for 25 min. FAMEs were formed through methylation by adding 2 mL of methylating reagent (325 mL 6.0 *N* HCl, 275 mL methanol), and vortexing and heating them to 80°C for 10 min. FAMEs were extracted from the aqueous phase into an organic phase using 1.25 mL extraction reagent (100 mL hexane, 100 mL methyl-tert butyl ether) tumbled for 10 min. Last, the aqueous phase was removed with a Pasteur pipette, washed with 3 mL of base wash (10.8 g NaOH, 900 mL distilled water) and tumbled for 5 min. Prior to chromatographic analysis, the organic phase containing FAMEs was transferred to glass vials and then analyzed using the Microbial Identification System (MIDI, Inc., Newark, DE USA). The output yielded sample-specific fatty acid (FA) peak responses, which were then used to estimate relative abundance of bacterial and fungal lipids. Relative abundance of bacterial lipids was estimated using branched-chain saturated (e.g., *iso* and *anteiso*), hydroxyl (OH), monounsaturated, and cyclopropyl FAs [55], whereas fungal lipid relative abundance was estimated using three lipid markers (18:2ω6, 18:1ω9c, and 18:3ω6c) [58, 59]. Estimates of relative fungal and bacterial lipid abundance were compared to determine relative differences in fungal and bacterial lipids between leaf species over the study. Microbial lipid profiles were compared to examine leaf species-specific differences in microbial assemblage composition over the study.

#### DNA extraction for molecular analyses of bacterial assemblages

To reduce potential bias resulting from using a single molecular technique, litter subsamples were collected and used for 3 separate molecular analyses: 1) ribosomal intergenic spacer analysis (RISA), 2) DGGE, and 3) bar-coded next-generation sequencing of 16S rRNA gene amplicons. Sequencing DGGE ribotype bands of the V3 region from the 16S rRNA gene coupled with bar-coded next-generation sequencing of 16S rRNA gene amplicons of the V4 region both provide taxonomic information but target different regions of the 16S rRNA gene to eliminate potential biases associated with targeting a single region. Bar-coded next-generation sequencing of 16S rRNA gene amplicons provides increased phylogenetic resolution compared to DGGE band sequencing and can be used to examine shifts in overall assemblage composition rather than only those of more abundant taxa. Techniques such as RISA, in addition to PLFA, also can be used to assess shifts in assemblage composition, but do so by targeting other cell components, e.g. fatty acids and intergenic spacer regions, thus reducing biases associated with a specific target region and allowing for processing of more samples than is feasible using sequencing alone. Here, genomic DNA was extracted from a 0.10-g litter subsample using a Qiagen genomic DNA extraction kit (Qiagen, Valencia, CA, USA). DNA was purified using cetyltrimethylammonium bromide (CTAB) extraction [60]. In some samples, particularly for day 0, extracted DNA was not sufficiently pure to serve as template for PCR. For these samples, we conducted additional genomic DNA purification using a combination of 80% formamide and 1M NaCl treatment to provide PCR-ready genomic DNA template [61]. This purification step has been tested with DNA extracted from many different environments and has not been observed to result in any loss of DNA or corresponding loss of diversity as assessed by DGGE. If the formamide step was deemed necessary by low PCR amplification using DNA templates derived from commercial kit extraction for a given sample date, then this method was applied to all samples on that date.

#### Rapid comparison of bacterial assemblages using RISA

RISA analysis was conducted as a rapid assay of bacterial assemblage composition among leaf packs over the study. RISA involved PCR amplification of bacterial internal transcribed spacer (ITS) regions and separating polymorphic ITS amplicons within a polyacrylamide gel matrix. PCR was conducted with a reaction volume of 10 μL containing GoGreen Master Mix (Promega, Madison, WI, USA), 1x Bovine Serum Albumin (BSA), nuclease free water, primers, and approx. 1–5 ng genomic DNA template, quantified spectrophotometrically with a NanoDrop ND-1000 (Thermo Fisher Scientific, Wilmington, DE, USA). Primers used for these reactions were the universal bacterial primers IRDYE 800-labeled ITSF (5'-GTCGTAACAAGGTAGCCGTA-3') [62] and ITSReub (5'-GCCAAGGCATCCACC-3') [62] at a final concentration of 0.20 μM. This primer set is not as susceptible as other primers to PCR biases such as those from substrate reannealing [63] and preferential amplification of shorter DNA templates [62]. A hot start PCR was used to prevent non-specific amplification, and the PCR products were robust. Amplification was done according to Fisher & Triplett [64] as follows: reaction mixtures were held at 94°C for 2 min, followed by 30 cycles of amplification at 94°C for 15 s, 55°C for 15 s, and 72°C for 45 s, and a final extension of 72°C for 2 min. We verified PCR products on a 1% agarose gel stained with ethidium bromide. Following verification of product yield and size, we separated amplicons in a 5.5% polyacrylamide gel matrix and images were recorded using a Li-Cor 4300 (Li-Cor Inc., Lincoln, NE, USA).

#### Identification of abundant bacterial taxa using DGGE

DGGE was used to allow comparison to previous studies and to assess taxon relative abundance [65] within litter over the study. Replicates from each sampling date were prepared using a 2-step process. First, genomic DNA extracted from leaf subsamples was used as a template in a PCR. Fifty μL reactions were conducted using a GoGreen Master Mix (Promega, Madison, WI, USA) that included Taq polymerase, dNTPs, and Mg^+2^-containing buffer (at 1x concentration). In addition, PCR reactions included 5 μL of 1:50 diluted DNA template, 1x BSA and 0.20 μM each of the universal bacterial primer set 518R (5′-ATTACCGCGGCTGCTGG-3′) [66] and 338F-GC (5’-CGCCCGCCGCGCCCCGCGCCCGTCCCGCCGCCCCCGCCCTCCTACGGGAGGCAGCAG-3’) [65]. This specific primer set was chosen to amplify the V3 region of the 16S rRNA gene, which can resolve bacterial taxa and produce comparable results to full-length (V1–V9) 16S rRNA gene sequence [67]. PCR conditions included 2-min of denaturation at 95°C followed by 30 cycles of 95°C for 1 min, 1 min of annealing at 55°C, and then 2 min of extension at 72°C [68]. Following PCR, an 8% polyacrylamide gel was poured containing a vertical gradient of formamide and urea at a final gradient concentration range of 45 to 55%. PCR products were loaded in the gel with 20 μL (~198–240 ng) per lane and electrophoresed for 15 h at 100 V and 60°C. Gels were then stained with ethidium bromide for 10 min and rinsed in deionized water for 15 min, after which bands were visualized using an AlphaImager HP gel documentation system (Alpha Innotech, San Leandro, CA, USA). Individual bands were considered distinct ribotypes [69]. Abundant rRNA gene amplicons for a given sampling time were visually identified, excised, and used as template in a subsequent PCR. All subsequent reactions were done in a total volume of 25 μL containing GoGreen Master Mix (Promega, Madison, WI, USA), primers 518R and 338F (without the GC clamp), and 2 μL of excised PCR product. All PCRs generated products without requiring further resolution of bands, and were sequenced using 518R (5 μM) and BigDye sequencing chemistry by the Lucigen Corporation (Middleton, WI). Unaligned sequences were compared to the GenBank nr/nt database using the BLASTn search algorithm at the National Center for Biotechnology Information (NCBI) to obtain the top ten nearest related bacterial taxa (≥95% similarity) based on 16S rRNA sequence identity. Previous studies reported that a portion of the16S rRNA gene amplicons, generated using universal 16S rRNA bacterial primers and isolated via DGGE, corresponded to plant 16S rRNA gene sequences [70, 71]. However, no mitochondrial or plastid sequences were obtained from our excised DGGE amplicons.

#### Bacterial assemblage characterization using paired-end sequencing of 16S rRNA gene amplicons

Replicate samples of each leaf species from a subset of incubation times (days 0, 32, and 128, representing early, mid-, and late-stages of breakdown, respectively) were used for next-generation sequencing of 16S rRNA gene amplicons to obtain a more comprehensive measure of bacterial assemblage diversity and composition. Amplification and sequencing of the V4 region of the 16S rRNA gene was performed using a modified method from Caporaso *et al*. [72]. Briefly, each sample was amplified using a 25-μL PCR reaction. Each reaction contained 12.5 μL KAPA HiFi HotStart ReadyMix (at 1x concentration)(Kapa Biosystems, Boston, MA, USA), 0.75 μL of the forward primer 515F (10 μM), 0.75 μL of the reverse primer 806R (10 μM), 2 ng DNA template, and PCR-grade water. Forward and reverse primers were modified according to Caporaso *et al*. [72] to include Illumina MiSeq flowcell adapter sequences, linker and pad regions, and a 12-bp Golay barcode on the reverse primers. Touchdown PCR conditions were as follows: 95°C for 2 min; 12 cycles of 98°C for 20 s, 61C for 30 s, decreasing 1°C at every cycle, and 72°C for 30 s; 20 cycles of 98°C for 20 s, 50°C for 30 s, and 72°C for 30 s; and 72°C for 10 min. PCR products were precipitated in 95% ethanol, re-suspended in 15 μL sterile molecular grade water, verified on a 1% agarose gel, and quantified using a Qubit fluorometer v2.0 (Life Technologies, Carlsbad, CA, USA). Following quantification, amplicons were pooled at equimolar concentrations and size selected using the E.Z.N.A. NGS Clean-IT kit (Omega Bio-Tek, Norcross, GA, USA) to remove primer dimers. The pooled, size-selected library was quantified using Qubit and sequenced using a final library concentration of 6.1 pM with a 30% PhiX spiked-in control. Paired-end sequencing was done using a 2x150 MiSeq reagent kit (Illumina, San Diego, CA, USA) on an Illumina MiSeq and involved 3 sequencing primers added [72]. Following sequencing, image analysis, base calling, and error estimation were done using MiSeq Reporter Software v1.1.6 (Illumina, San Diego, CA, USA).

### Data analyses

We used a mixed effects model to test the fixed effects of date, leaf species, and the date-species on litter breakdown and microbial assemblage composition. This model also included the random terms of block nested within date and the interaction of block nested within date and leaf species. To confirm the degree of difference in streamwater conditions among runs, we also compared initial current velocity and water depth using the above model. Residual plots were examined to check the assumptions of linear modeling. Current velocity at time of removal and relative fungal biomass were square-root transformed prior to analysis. Tukey’s post-hoc tests were used to compare mean initial current velocity among rus and mean AFDM remaining among leaf species [73].

RISA gel images were analyzed using BioNumerics Software v5.0 (Applied Maths, Kortrijk, Belgium) to quantify bacterial assemblage similarity. Bands were used to create a presence-absence matrix for further analysis. Bands were defined relative to the highest band density on that pattern, where all bands, with a density >10% of the highest band density, were selected for further analyses. Similarities between band presence-absence fingerprints were calculated using Jaccard’s coefficient, and cluster analysis (Ward’s method) [74] was then used to create dendrograms to visualize bacterial assemblage similarity [71].

Paired-end, bar-coded 16S rRNA gene sequences were aligned using PANDAseq [75], and aligned sequences (“contigs”) were analyzed further using the QIIME pipeline [76]. Sequences were sorted by barcode, and all reads ≥75 bp and with a Q-score ≥ 20 were included in all downstream analyses. Using QIIME, operational taxonomic units (OTUs) were picked at 97% similarity using UCLUST [77], and a representative set of sequences was generated based on the most abundant sequence observed for an OTU. This representative set of sequences was aligned using PyNAST [78] against the Greengenes database [79], and taxonomy was assigned using RDP classifier [80]. Chimeric sequences were removed using ChimeraSlayer [81], and sequences classified as chloroplast (not including Cyanobacteria) were filtered out with QIIME. Alpha diversity metrics were calculated for bar-coded next-generation 16S rRNA gene sequence data, including OTU abundance, Chao1 richness estimates, phylogenetic distance, and Shannon’s diversity [82, 83]. Beta diversity was estimated by calculating weighted and unweighted UniFrac distances [84]. An even sampling depth of 4,262 sequences was used to rarify all samples prior to all diversity estimations. The mixed model mentioned above was used to test the fixed effects of leaf species and date on alpha diversity metrics and bacterial taxa abundance. Phylogenetic distance estimates were Box-Cox transformed prior to analysis [85], and a Yeo-Johnson transformation was applied to Flavobacteria relative abundance data to satisfy linear modeling assumptions [86]. Relative abundance of Proteobacteria classes (α,β, and γ) also was transformed using a Yeo-Johnson transformation to satisfy normality [86], and we used a Kruskal-Wallis test [87] to test for the effects of leaf species and date on relative abundance of the Proteobacterial classes. We also used a Kruskal-Wallis test [87] to test for effects of leaf species and date on the relative abundance of *Acidobacteria*, *Bacteroidetes*, *Verrucomicrobia*, and *Sphingobacteria*. Jackknifed cluster analysis was done on bar-coded next-generation 16S rRNA gene sequence data based on weighted and unweighted UniFrac distances using unweighted pair group method with arithmetic mean (UPGMA) [88].

Overall lipid profiles and bacterial assemblage composition (both RISA patterns and bar-coded sequences) were compared for each leaf species over time using Analysis of Similarity (ANOSIM) [89]. For these comparisons, a Bray-Curtis dissimilarity matrix [90] was calculated for lipid and RISA profile comparisons, and unweighted UniFrac distances were used for comparison of bar-coded next-generation 16S rRNA gene sequence data among samples. An alpha level of 0.05 was used for all statistical analyses.

### Nucleotide sequence accession number

All sequences obtained from extracted DGGE bands and bar-coded next-generation sequencing were submitted to the NCBI Sequence Read Archive (SRA) under the study accession number SRP033423.

## Results

### Environmental conditions

Streamwater pH and dissolved oxygen measurements on day 1 indicated the stream was acidic (pH = 4.33), but well oxygenated (8.65 mg/L, 88% saturation). Initial measurements for all runs on day 0 revealed a mean (± SE) water depth of 0.16 ± 0.01 m and a mean current velocity of 0.07 ± 0.01 m/s. Initial water depth among runs did not differ (*p* = 0.074), whereas initial current velocity varied among runs (*p* = 0.032). Tukey’s pairwise comparisons of initial current velocity among runs indicated that the run sampled on day 2 was more similar to the runs sampled on days 32 and 64 than any other runs, but given that leaf litter microbial assemblage data on day 2 was more similar to days 0, 4, and 8, this difference in current velocity likely had little effect on the leaf litter microbial assemblage relative to sampling date. Water temperature during the 128-d study decreased from a mean of 15.2°C (day 1, 4 Jan) to a minimum of 3.7°C (day 44, 16 Feb) and reached a maximum of 30.3°C by day 128 (12 May), with a mean temperature of 13.4°C over the incubation period. Mean water depth at individual leaf packs when retrieved was 0.17 (± 0.01) m, which did not differ between leaf species (*p* = 0.791). Mean current velocity immediately upstream of retrieved leaf packs was 0.07 (± 0.01) m/s, which also did not differ between species (*p* = 0.118). Mean water depth at leaf packs decreased significantly (*p* = <0.001) from 0.31 m on day 1 to 0.09 m on day 128. Mean current velocity also varied over time (*p* = 0.003) with highest velocity on day 4 (0.12 m/s) and lowest on day 8 (0.02 m/s).

### Litter breakdown

**Fig 1:**
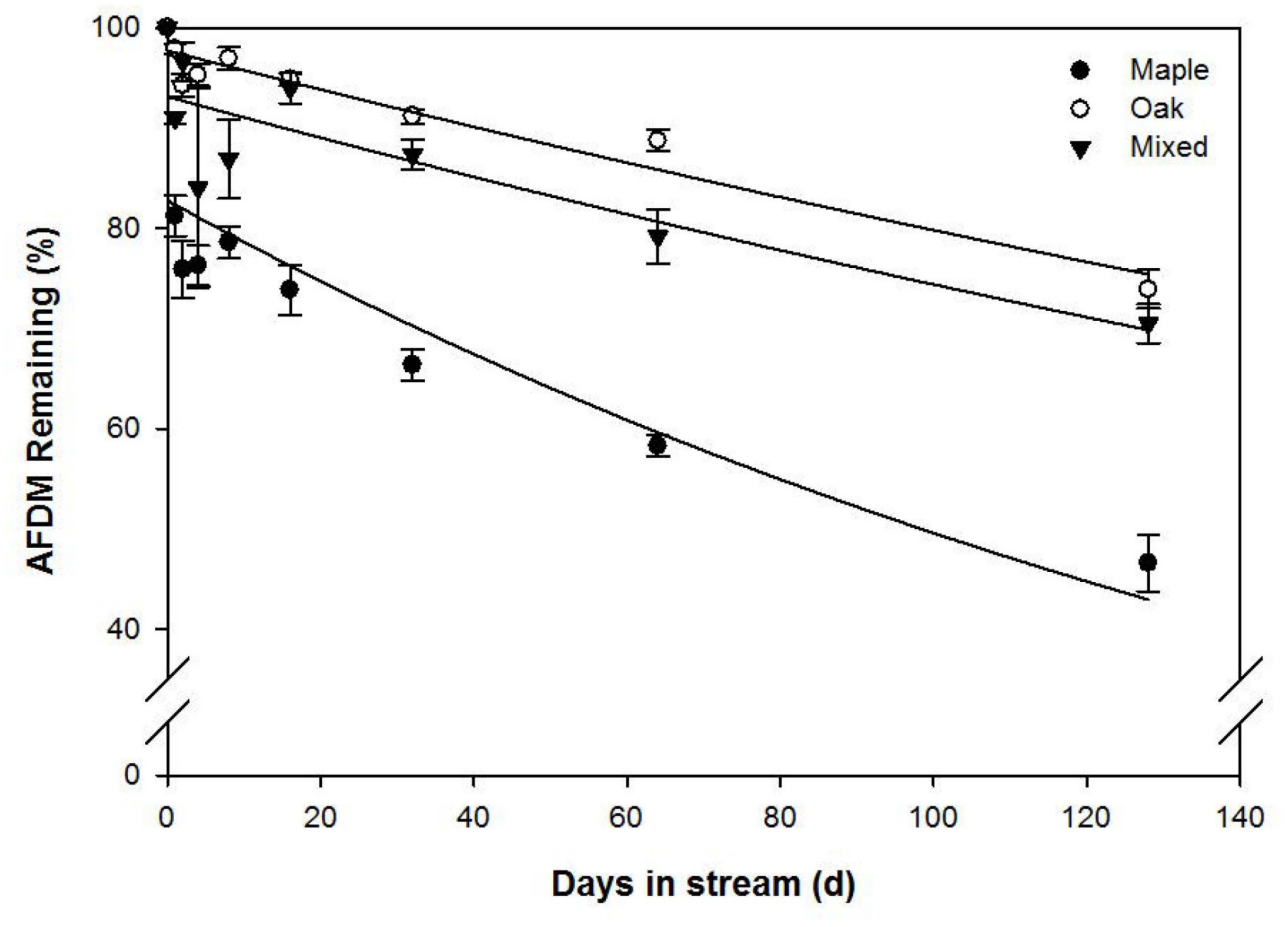
Mean (± 1SE) ash free dry mass (AFDM) remaining over time during breakdown of red maple (closed circles), water oak (open circles), and mixed litter (inverted solid triangle) leaf packs incubated for 128 d in Kings Mill Creek, GA, USA.

Over the study, mean litter breakdown rates (*k*) for red maple (hereafter maple), water oak (hereafter oak), and mixed litter were 0.075, 0.026 and 0.033 *d* ^-1^, respectively (Fig 1). Breakdown varied significantly between leaf species (*p*<0.001). Maple packs contained significantly less litter over time than both oak and mixed-species packs. Mixed-species breakdown was significantly faster than oak and significantly slower than maple (Fig 1). The exponential decay model explained 85.6, 94.8, and 87.9% of the variation in maple, oak, and mixed litter breakdown, respectively (Fig 1). Maple AFDM decreased rapidly from 100 to 81.2% remaining after 1 day’s incubation. In contrast, oak showed little AFDM change over the same interval (~2% loss), and mixed litter decreased from 100 to 91.0%. After 128 d, maple had 46.6% AFDM remaining, compared to 73.9% remaining for oak and 70.5% for mixed litter packs.

### Microbial assemblage characterization

**Fig 2:**
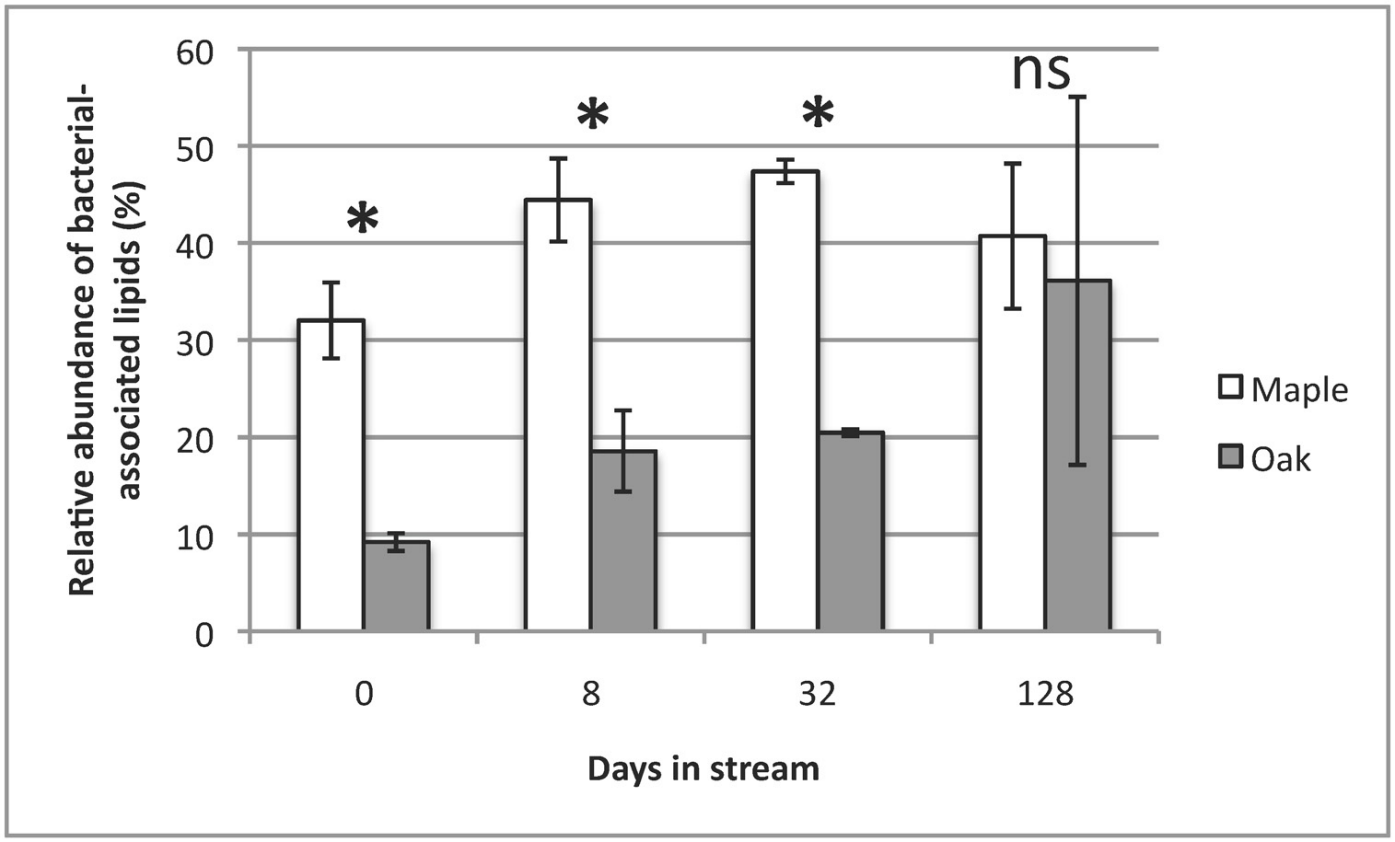
Mean (± 1SE) % relative abundance of bacterial lipid markers of red maple and water oak leaf packs over a 128-d incubation in Kings Mill Creek, GA, USA. (* = *p*<0.001, ns = not significantly different).

**Fig 3:**
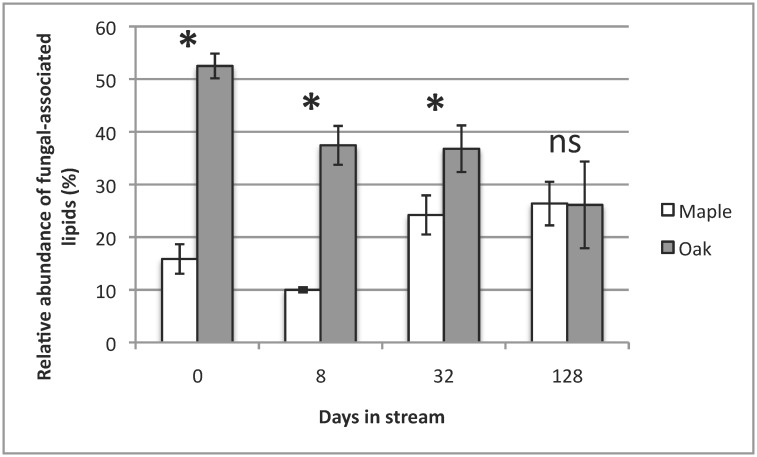
Mean (± 1SE) % relative abundance (%) of fungal lipid markers of red maple and water oak leaf packs over the 128-d incubation In Kings Mill Creek, GA, USA. (* = *p*<0.001, ns = not significantly different).

Relative bacterial and fungal lipid abundance, estimated by FAME analysis, differed significantly between maple and oak on all dates except day 128 (Figs 2 and 3). Overall, bacterial lipid relative abundance on maple was higher than oak (*p*<0.001, Fig 2), whereas oak showed higher fungal lipid relative abundance than maple (*p*<0.001, Fig 3). Fungal lipid relative abundance on oak tended to decrease over the incubation, whereas bacterial lipid abundance steadily increased (Fig 3). Bar-coded next-generation sequencing yielded 195,835 paired-end sequences with quality scores >20 and a mean of 11,311 sequences per sample. The number of observed bacterial OTUs, based on bar-coded next-generation sequence data, increased over time for both species, ranging from ~337 OTUs on day 0 to ~979 OTUs on day 128 for maple and ~463 OTUs on day 0 to ~775 OTUs on day 128 for oak. There was no effect of leaf species on any bacterial alpha diversity measure (*p*>0.05 for all metrics), but all 4 alpha diversity metrics varied over time (*p*<0.001, Table 1). However, there was no species-date interaction for any bacterial alpha diversity metric (*p*>0.05 for all metrics, Table 1).

**Table 1:** Comparison of alpha diversity metrics calculated for maple and oak leaf litter bacterial assemblages from paired-end sequencing results following 0, 32, and 128 days instream incubation at an even sampling depth of 4260 sequences.

Treatment		Alpha Diversity Metric			
	Day	Chao1	OTUs	Phylogenetic Distance	Shannon's Index
Maple	0	1043.41 ± 34.44 ^c^	337.10 ± 25.40 ^c^	18.96 ± 0.30 ^d^	4.45 ± 0.35 ^c^
	32	1480.27 ± 269.69 ^bc^	558.33 ± 96.37 ^bc^	37.65 ± 4.33 ^bc^	5.96 ± 0.67 ^abc^
	128	2209.96 ± 209.93 ^a^	978.97 ± 124.12 ^a^	57.44 ± 8.21 ^a^	7.73 ± 0.58 ^a^
Oak	0	1446.00 ± 95.58 ^bc^	463.30 ± 106.95 ^c^	22.32 ± 2.11 ^cd^	5.28 ± 0.80 ^bc^
	32	1608.40 ± 27.06 ^bc^	604.97 ± 5.62 ^bc^	33.62 ± 1.96 ^bcd^	6.58 ± 0.09 ^ab^
	128	1985.74 ± 160.85 ^ab^	774.93 ± 69.05 ^ab^	48.73 ± 4.81 ^ab^	6.73 ± 0.36 ^ab^

Superscript letters beside metric values represent Tukey’s multiple comparison groupings for each metric (α = 0.05).

**Fig 4:**
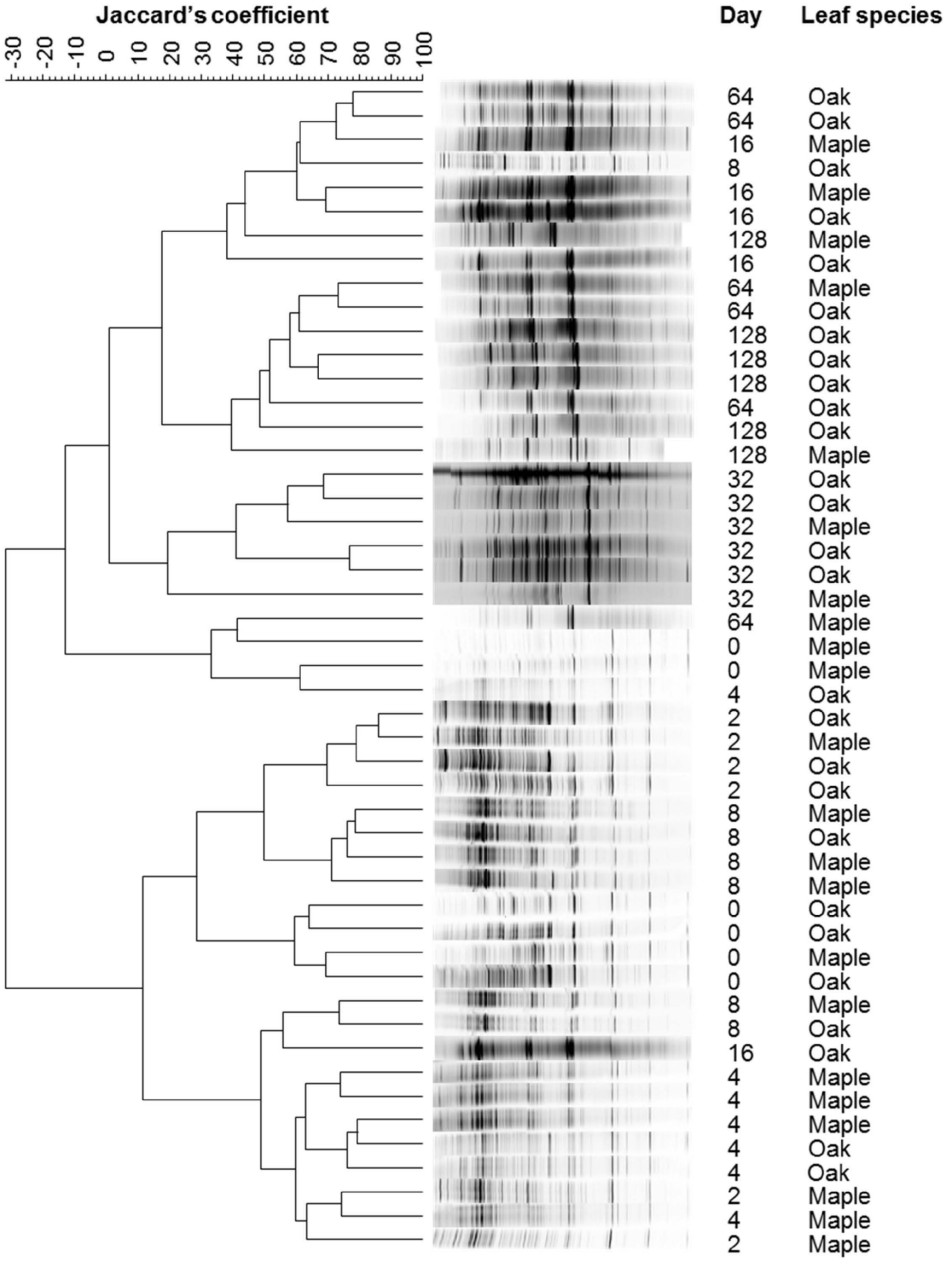
Dendrogram of ribosomal intergenic spacer analysis (RISA) electropherograms displaying bacterial assemblage similarities calculated using Ward’s method based on Jaccard’s similarity coefficient.

**Fig 5:**
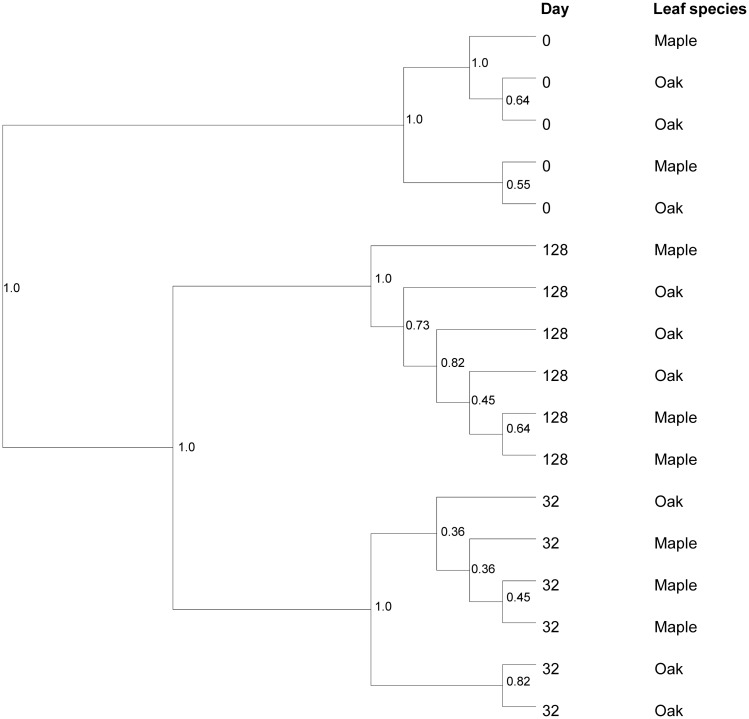
Unifrac-based unweighted pair group method with arithmetic mean (UPGMA) clustering of maple and oak sequences following 0, 32, and 128 days of instream incubation. Values at nodes represent level of clustering support expressed as a decimal ranging from 0 to 1.0.

Analysis of bacterial assemblage composition using RISA showed little dependence on leaf species (*p* = 0.076); in contrast, composition varied strongly by date (*p*<0.001, Fig 4). PLFA also indicated high variation in lipid profiles over time for both maple and oak (*p* = 0.001 and 0.005, respectively). Cluster analysis of RISA data clearly defined 3 temporal groupings of bacterial assemblages, pre-immersion, early breakdown, and later breakdown assemblages (Fig 4). Jackknife-based UPGMA clustering of weighted and unweighted UniFrac distances, calculated from next-generation 16S rRNA gene sequence data, both suggested structuring of bacterial assemblages into 3 temporal groupings. There was no overall effect of leaf species on bacterial assemblage structure (unweighted *p* = 0.525; weighted *p* = 0.605) based on next-generation 16S rRNA gene sequence data, although as with PLFA, assemblage structure varied strongly with date (*p*<0.001) (Fig 5).

Sequenced DGGE bands yielded the identity of several common bacterial taxa within litter (S2 Fig and S3 Fig). Bacteria on maple included the genera *Ralstonia* (*Burkholderiales*, *β-Proteobacteria*; day 0), *Sphingopyxis* (*Sphingomonadales*, *α-Proteobacteria*; days 1 and 32), *Delftia* (*Burkholderiales*, *β-Proteobacteria*; day 4), *Herbaspirillum* (*Burkholderiales*, *β-Proteobacteria*; day 4), *Nitrosospira* (*Nitrosomonadales*, *β-Proteobacteria*; day 8), and *Collimonas* spp. (*Burkholderiales*, *β-Proteobacteria*; days 16, 64, and 128), with sequence identities to GenBank matches ranging from 95 to 100%. *Citrobacter* (*Enterobacteriales*, *γ-Proteobacteria*) occurred on oak on all dates. Genera from 4 other ribotypes also were abundant, including *Sphingomonas* (*Sphingomonadales*, *α-Proteobacteria*; day 1), *Aquabacterium* (*Burkholderiales*, *β-Proteobacteria*; day 8), *Sphingopyxis* (*Sphingomonadales*, *α-Proteobacteria*; day 0), and *Thiobacillus* (*Hydrogenophilales*, *β-Proteobacteria*; day 128), with sequence identities to GenBank matches ranging from 95 to 100%. Total bacterial ribotype richness was higher for maple than oak, with 21 distinct ribotypes on maple (S2 Fig) and 18 on oak (S3 Fig). The highest ribotype richness for maple (14) was on day 32, whereas richness on oak was highest on days 0 and 1 (10 and 16 ribotypes, respectively).

**Fig 6:**
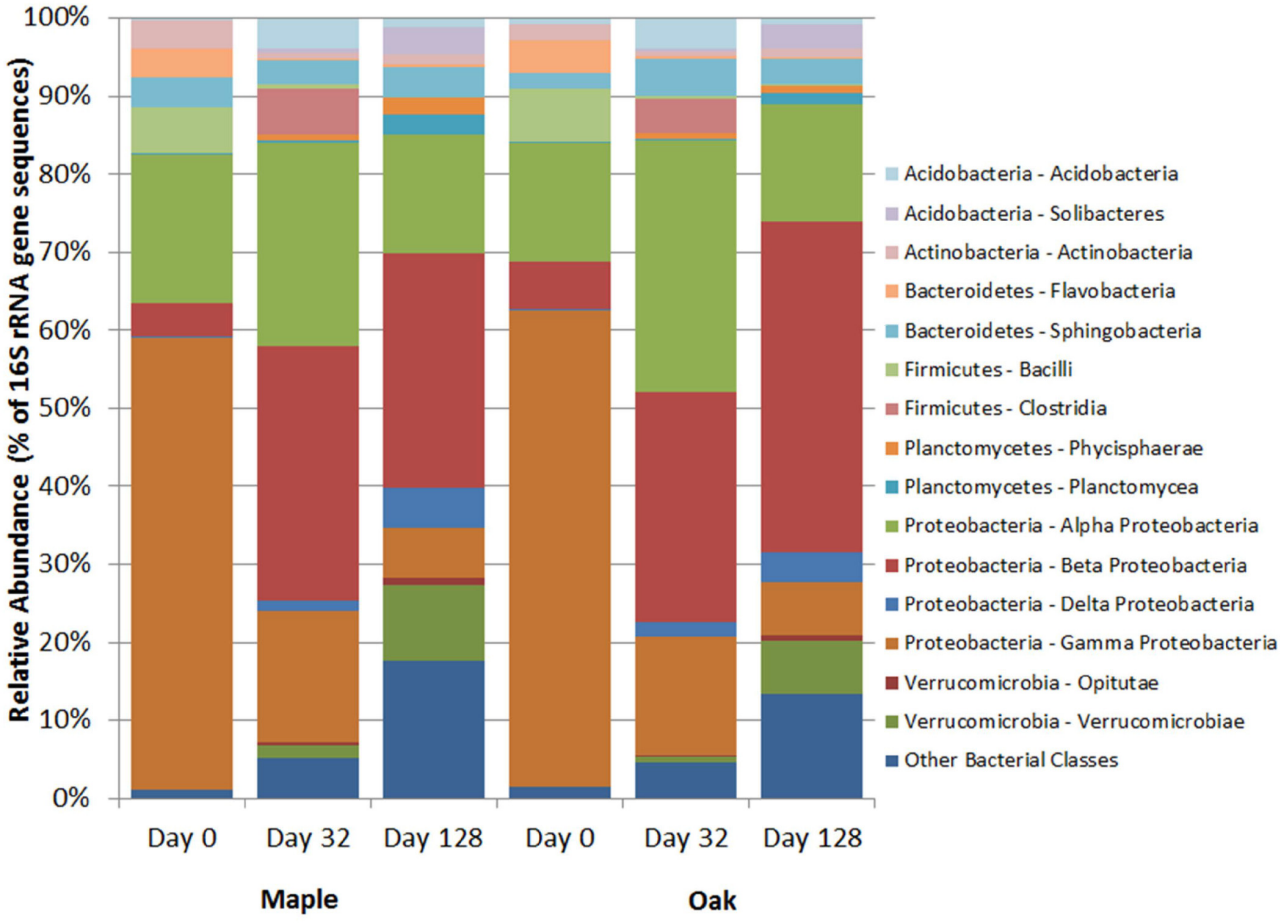
Relative abundance of class- and phylum-level bacterial taxa. Data derived from bar-coded next-generation sequencing of 16S rRNA gene amplicons and represent taxa affiliation summaries for both leaf species (maple and oak) following 0, 32, and 128 days in stream incubation. Classes representing <0.5% in all samples were grouped and represented as “Other” for their given phylum, whereas phyla representing <0.5% of all sequences were grouped as “Other Bacterial Classes”.

A summary of taxa obtained from next-generation 16S rRNA gene sequencing is presented in Fig 6 for both the phylum and class levels. The phylum *Proteobacteria* dominated both maple and oak over the study, although the dominant class varied with date. Prior to instream incubation, maple and oak both contained mostly γ-*Proteobacteria* (54.0 and 56.6%, respectively) and also α-*Proteobacteria* (17.8% maple; 14.1% oak). Many of the γ-*Proteobacteria* came from the families *Aeromonadaceae*, *Enterobacteriaceae*, and *Pseudomonadaceae*. Abundance of α*-Proteobacteria* before incubation was mostly taxa from the orders *Rhizobiales* and *Sphingomonadales*.

The relative abundance of α- and β-*Proteobacteria* did not differ between leaf species (*p* = 0.962 α- and *p* = 0.923 β-*Proteobacteria*) but varied over time (*p* = 0.001 α- and *p* = 0.053 β-*Proteobacteria*). After 32 days, most *Proteobacteria* were from the α- and β-*Proteobacteria* classes, which increased in relative abundance during this interval. Many of the β-*Proteobacteria* sequences were within the order *Burkholderiales* (particularly the family *Oxalobacteraceae*). For α*-Proteobacteria*, the relative abundance of *Sphingomonadales* increased after 32 days, as did the orders *Caulobacterales* (mainly *Caulobacteraceae*) and *Rhodospirillales* (mostly *Rhodospirillaceae* and some *Acetobacteraceae*). By 128 days, the relative abundance of β-*Proteobacteria* sequences remained steady (25.7% maple, 37.8% oak) compared to those at day 32 (30.3% maple, 25.6% oak), but the number of sequences from α-*Proteobacteria* decreased compared to the abundance observed at day 32 (from 24.2 to 12.9% in maple, 27.9 to 13.4% in oak). The abundance of sequences that affiliated with the γ-*Proteobacteria* did not differ between leaf species (*p* = 0.630) but declined over time (*p* = 0.005). Specifically, between days 0 and 32 γ-*Proteobacteria* decreased from 54.0 to 15.8% in maple and 56.6 to 13.2% in oak, with most γ-*Proteobacteria* sequences on day 32 being in the family *Pseudomonadaceae* (75.3% maple, 65.2% oak). Last, relative abundance of γ-*Proteobacteria* declined from 15.8% (maple) and 13.2% (oak) on day 32 to 5.5% and 6.2%, respectively, on day 128 (Fig 6).

Other litter bacterial phyla varying over time included *Acidobacteria*, *Bacteroidetes*, and *Verrucomicrobia*. Overall relative abundance of *Acidobacteria* sequences did not differ between leaf species (*p* = 0.700) but increased over time (*p* = 0.007), from 0.4% (maple) and 1.1% (oak) on day 0 to 5.3% (maple) and 4.4% (oak) on day 128, many from the family *Solibacteraceae*. Relative abundance of *Bacteroidetes* sequences also did not differ between species (*p* = 0.773) but decreased over time (*p* = 0.023). Most *Bacteroidetes* sequences were from the class *Sphingobacteria*, whose abundance did not vary over the study (*p* = 0.574); in contrast, abundance of *Flavobacteria* sequences decreased (*p* = 0.002) over the study from 7.2 to 5.0% (maple) and 5.9 to 3.7% (oak). Relative abundance of *Verrucomicrobia* (mainly from the order *Verrucomicrobiales*) did not differ between species (*p* = 0.290) and increased over time (*p* = 0.001), from 0.1% for both species pre-incubation to 11.8% and 7.9% in maple and oak, respectively, after 128 days.

## Discussion

Breakdown rates strongly differed between leaf species in our study, with observed rates being faster for red maple than water oak, a result consistent with previous work [1]. This difference in breakdown likely reflects intrinsic physicochemical differences between oak and maple leaf species with higher lignin content, a thicker cuticle, and increased tannins likely contributing to slower breakdown for oak [1, 17, 47, 91]. PLFA results from our study were similar to others [38] suggesting that time of incubation is the main determinant of microbial assemblage structure, as microbial lipid profiles of both species varied strongly with incubation time. However, there were clear species-level differences in microbial assemblages. The higher relative bacterial lipid abundance on maple (vs. oak) could have resulted from oak having a smaller leaf surface area available for colonization by bacteria compared to maple, or also having higher tannin and lignin content, as well as a thick waxy cuticle, thus reducing bacterial colonization because of higher refractory materials. Das *et al*. [38] also observed increased bacterial lipid abundance on sugar maple compared to oak, and cited the availability of colonizable physical leaf substrate as a plausible reason for the difference between species. In addition, the faster breakdown of maple may increase nutrient availability on leaf surfaces and thus stimulate bacterial growth [32]. Alternatively, oak showed higher relative fungal lipid abundance than maple on most sampling dates, possibly because fungi are more capable of colonizing and degrading lignin-rich oak than bacteria [92, 93], potentially allowing fungi to dominate for a longer period under such conditions. In contrast, increased nutrient availability on maple with lower lignin than oak may facilitate bacterial colonization, thus leading to increased bacterial lipid abundance over time. Initial lignin concentration has been indicated as a key factor in controlling breakdown by reducing available C in litter [91]. In our study, relative abundance of fungal lipids on oak did decrease over the 128-d incubation (Fig 3), so increased bacterial lipid relative abundance may have resulted from increased nutrients for bacteria following fungal colonization and conditioning [32].

Decreased maple-oak differences in bacterial and fungal lipid relative abundance during later breakdown (e.g., on day 128) also could be attributable to plant compounds (e.g. tannins, phenolics, etc.) present in higher quantities during early breakdown that leached or were otherwise diminished over time. Canhoto & Graça [94] demonstrated the inhibitory effects of secondary compounds, such as tannic acid, on fungal growth. In that study, decreased growth of four aquatic hyphomycete taxa occurred following addition of increasing concentrations of tannic acid and eucalyptus oils. In our study, chemical differences between maple and oak would likely be at their highest during initial incubation, before significant leaching; thus, our observations of the most extreme differences between maple and oak microbial lipid abundance occurring during initial breakdown are consistent with this mechanism. This equalizing of fungal and bacterial lipid relative abundance over time was also observed by Chapman et al. [95] using mixed and single species leaf litter. The decreased difference in microbial lipid relative abundance between maple and oak over time also may indicate increased bacterial colonization on oak following fungal conditioning, permitting colonization by litter-associated bacteria previously incapable of colonizing oak leaf surfaces, possibly by increasing available surface area or through development of fungal hyphae. The ability of fungal presence to facilitate bacterial colonization has been suggested [30], and higher bacterial colonization in response to increased surface area and available organic matter has been shown[96].

Maple and oak differed in initial bacterial lipid relative abundance, but both species showed similar bacterial alpha diversity overall based on next-generation sequencing data. This result would suggest that although initial leaf physicochemical differences may affect microbial lipid accumulation rates, these differences do not appear to affect diversity of taxa colonizing litter. RISA and next-generation sequencing results indicated that bacterial assemblage structure was more influenced by incubation time than by leaf species. Taken together, these results and our microbial lipid profiles support the results of Das *et al*. [38] who also found time was more important than leaf species in structuring fungal, bacterial, and actinomycete assemblages on litter.

DGGE analysis of bacterial composition showed similarities in dominant taxa (e.g. *Sphingopyxis*) on both leaf species. Both next-generation sequencing and DGGE showed the phylum *Proteobacteria* to be the dominant phylum present during leaf breakdown Dominant bacterial orders (i.e. *Sphingomonadales* and *Burkholderiales*) identified by sequencing of DGGE bands also were represented in next-generation sequencing data. Similar to the next-generation sequencing results, most *β*-*Proteobacteria* identified using DGGE were from the order *Burkholderiales*. Next-generation sequencing also identified many other taxa not observed using DGGE. Using these two methods allowed us to identify dominant taxa for all time points using DGGE and to then select a subset of specific time points for 16S rRNA gene sequencing to obtain a more comprehensive view of bacterial composition during breakdown. It is important to note that by comparing DGGE- and next-generation 16S rRNA gene sequence data, DGGE underrepresented taxa richness (and alternatively, next-generation sequencing overestimated taxa richness to some degree), so bacterial taxa observed in DGGE analysis of maple and oak leaves likely are the more dominant taxa at their respective dates of incubation. Next-generation 16S rRNA gene sequence data showed much higher bacterial taxon richness exists within these bacterial assemblages.

In general, prior to immersion the phyllosphere of both maple and oak leaves was dominated by α- and γ-*Proteobacteria*, and secondarily by β-*Proteobacteria*. In a study comparing terrestrial phyllosphere composition among several angiosperm and gymnosperm species, Redford *et al*. [97] noted a similar phyllosphere composition from these families. By examining litter bacterial assemblages at various time points before and during instream breakdown, our study is the first to document taxonomic shifts in bacterial assemblage composition both during the transition from the terrestrial phyllosphere to the aquatic environment as well as over time during instream incubation. The observed increase in α- and β-*Proteobacteria* likely occurs in response to the terrestrial-to-aquatic transition. Both α- and β-*Proteobacteria* are dominant groups within freshwater sediment assemblages [98, 99] and have been shown to dominate detrital aggregates in lentic systems [100].

Observed increases in abundance of bacteria within the orders Burkholderiales (family Oxalobacteraceae), Sphingomonadales (family Sphingomonadaceae), Caulobacterales (family Caulobacteraceae), and Rhodospirillales (families Acetobacteraceae and Rhodospirillaceae) as breakdown proceeds suggests the increased role of these taxa in progressive degradation of maple and oak. Each of these orders contain members with degradative, diazotrophic, and oligotrophic attributes that suit their colonization and use of submersed, degrading leaf litter, and many have been implicated in organic matter decomposition [101, 102]. Members of the more abundantly observed families (Oxalobacteraceae, Sphingomonadaceae, Caulobacteraceae, Acetobacteraceae, and Rhodospirillaceae) are common heterotrophic bacteria, and many are known from fresh water [103–105]. The family Oxalobacteraceae includes taxa that use organic compounds as an energy source as well as diazotrophic members [103]. Sphingomonadaceae is a nutritionally diverse family as well and is known for its degradative abilities [103]. Members of the family Caulobacteraceae are chemoorganotrophs and oligotrophs, which are capable of using organic C and surviving in low-nutrient environments [103]. Most genera of the family Rhodospirillaceae are photoheterotrophs often using organic compounds as a C source [103]. In our study, many sequences identified as members of the families Caulobacteraceae, Sphingomonadaceae, Oxalobacteraceae, and Rhodospirillaceae increased in abundance after 32 days of incubation and then decreased by day 128. Such dynamics would suggest 1) an initial increase in readily accessible organic material after the first month of instream incubation and leaching, and 2) a subsequent decrease in readily accessible organic matter, which, in turn, causes decreased abundance of these taxa.

Our results demonstrated that fast breakdown leaf species (i.e., red maple) were more readily colonized by bacteria, whereas slower degrading species (i.e., water oak) were initially dominated by higher fungal lipid abundance but supplanted by increasing relative abundance of bacterial lipids over time. As litter leachate rates temporally decrease and microbial conditioning increases available leaf surface area, colonization by additional bacterial species on oak may become less constrained by leaf physicochemical differences and more easily colonized by bacteria. Over time, as bacterial lipids accumulate on oak, they eventually reach a level similar to that of maple, whose leachate is more easily dispersed and is more readily colonized. Overall, our results suggest differences in leaf physicochemistry may affect the rate at which bacteria can colonize leaf litter, but these conditions do not play as great a role structuring bacterial assemblages of litter as does time of incubation.

Given the predominant role of incubation time in structuring leaf litter microbial assemblages, future studies could focus on investigating the role of seasonal and annual variability on litter microbial assemblage composition and turnover. In addition, the effect of incubation time on composition may vary with current velocity given its effect on litter leaching rate [106]. Future leaf breakdown studies conducted over a wide array of current velocity regimes (e.g., lotic to lentic environments) could explore the degree to which flow conditions modify the hierarchical effect of incubation time and leaf physicochemistry on structuring leaf litter microbial assemblages.

## Supporting Information

S1 Fig Diagram illustrating leaf pack arrangement within a single run unit. A = red maple, B = water oak, C = mixed litter.
(TIF)

S2 Fig Denaturing gradient gel electrophoresis (DGGE) analysis of red maple leaf pack bacterial assemblage. Upper case letters indicate sequenced ribotypes. (Ribotype key: A = *Delftia*, B = *Sphingopyxis*, C = *Herbaspirillum*, D = *Nitrosospira*, E = *Ralstonia*, F = *Collimonas*).
(TIFF)

S3 Fig Denaturing gradient gel electrophoresis (DGGE) analysis of water oak leaf pack bacterial assemblage. Upper case letters indicate sequenced ribotypes. (Ribotype key: B = *Sphingopyxis*, G = *Sphingomonas*, H = *Aquabacterium*, I = *Citrobacter*, J = *Thiobacillus*).
(TIFF)
