# Supplementary figures and images for: Litter Breakdown and Microbial Succession on Two Submerged Leaf Species in a Small Forested Stream
Source: PLoS One. 2015 Jun 22;10(6):e0130801. doi: 10.1371/journal.pone.0130801 (PMC4476575; doi:10.1371/journal.pone.0130801)

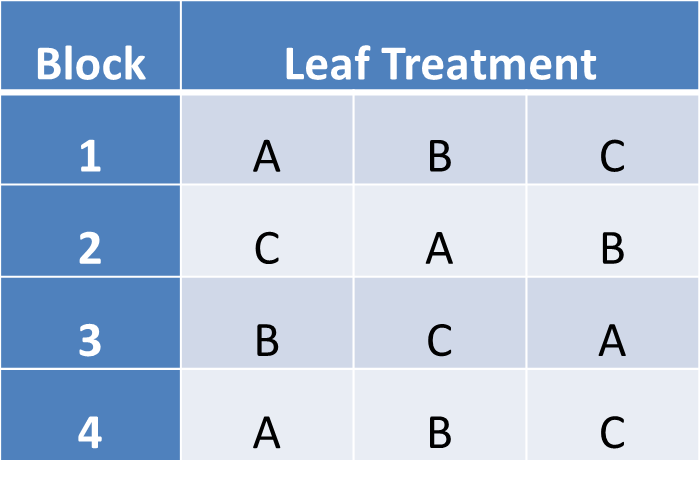

Supplement: S1 Fig — A = red maple, B = water oak, C = mixed litter. (TIF) [file pone.0130801.s001.tif]

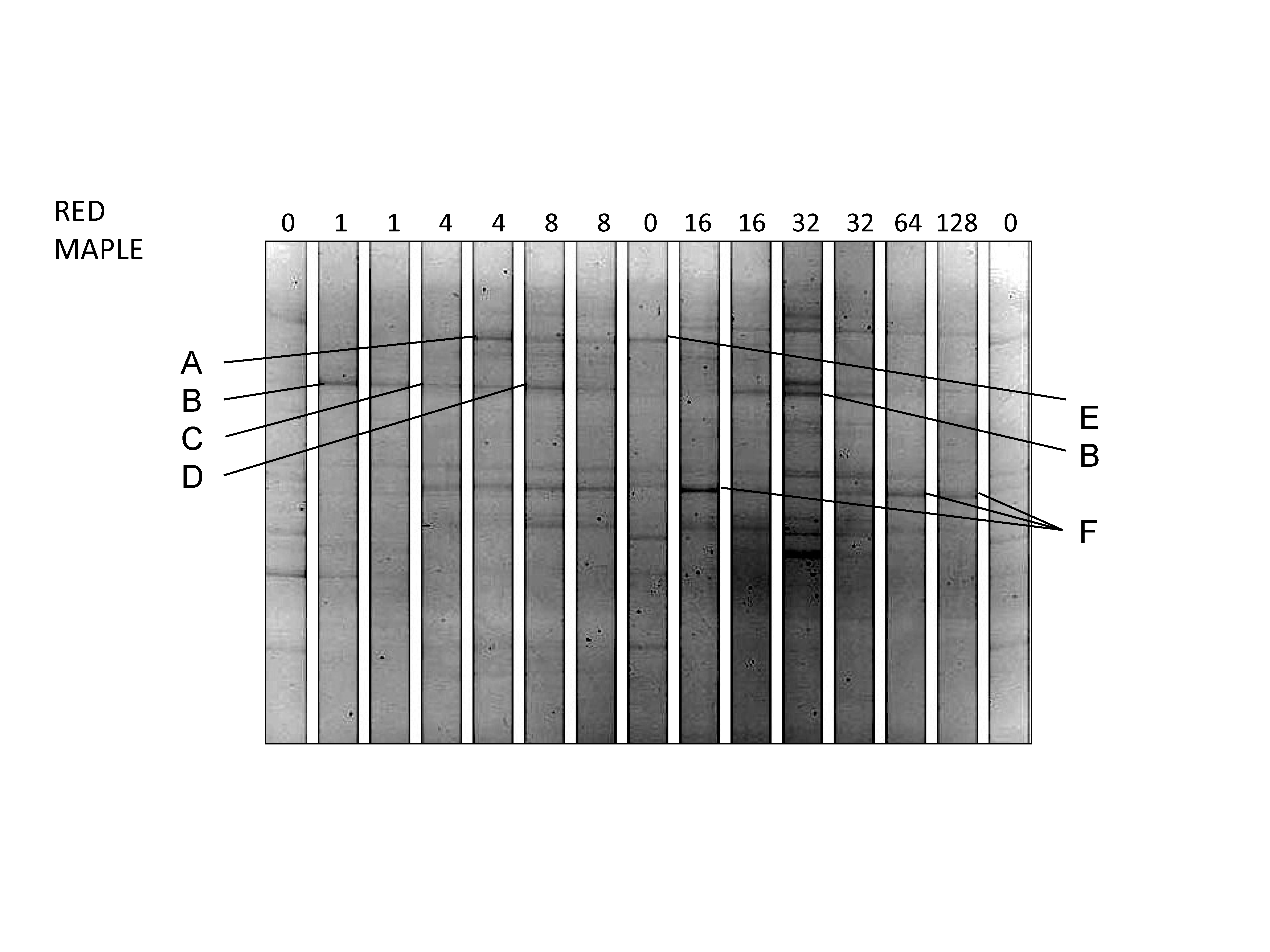

Supplement: S2 Fig — Upper case letters indicate sequenced ribotypes. (Ribotype key: A = Delftia, B = Sphingopyxis, C = Herbaspirillum, D = Nitrosospira, E = Ralstonia, F = Collimonas). (TIFF) [file pone.0130801.s002.tiff]

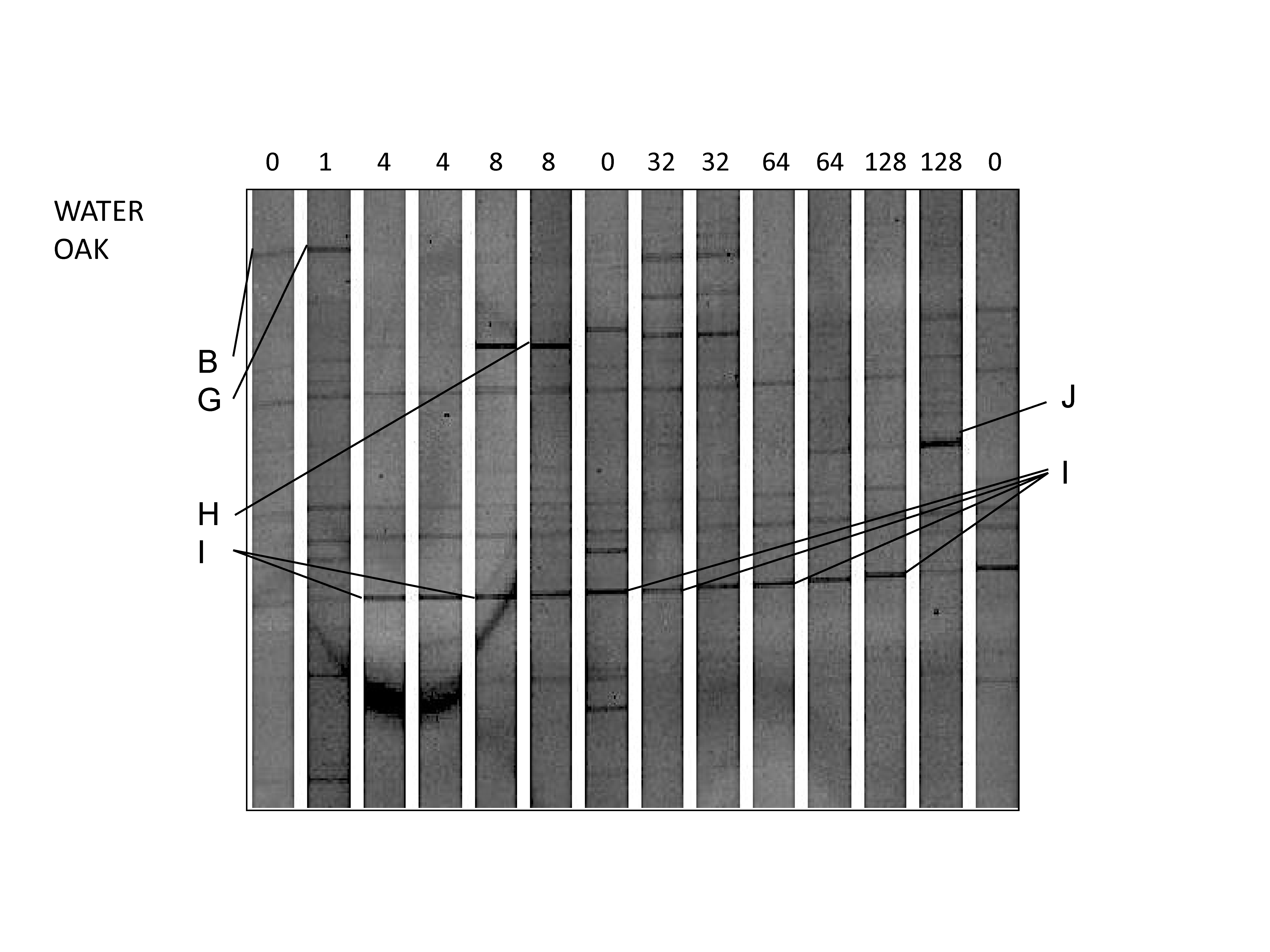

Supplement: S3 Fig — Upper case letters indicate sequenced ribotypes. (Ribotype key: B = Sphingopyxis, G = Sphingomonas, H = Aquabacterium, I = Citrobacter, J = Thiobacillus). (TIFF) [file pone.0130801.s003.tiff]
